# Supplementary material for: Deciphering the interactome of Ataxin-2 and TDP-43 in iPSC-derived neurons for potential ALS targets
Source: PLoS One. 2024 Dec 31;19(12):e0308428. doi: 10.1371/journal.pone.0308428 (PMC11687654; doi:10.1371/journal.pone.0308428)
Supplement: S1 File — (DOCX) [file pone.0308428.s003.docx]

**Figure 1A:**


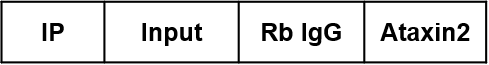


**150Kda**

**50Kda**


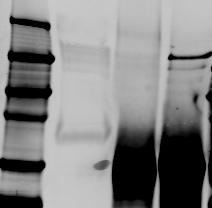

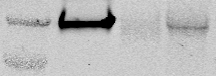


**WB:TDP-43**

**WB:Ataxin2**

**Figure 1A original:**

**Upper panel**


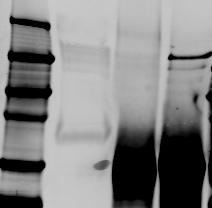


**WB:Ataxin2**

**Lower Panel**


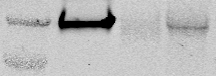


**WB:TDP-43**

**Figure 1B:**


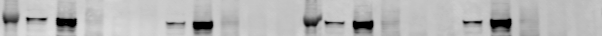

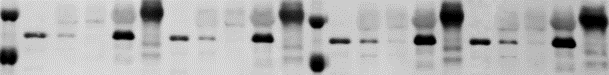

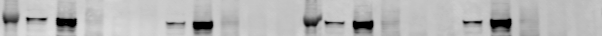

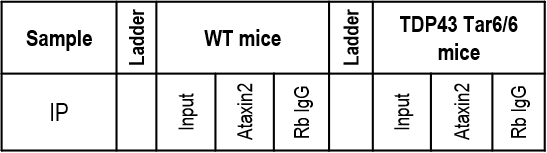

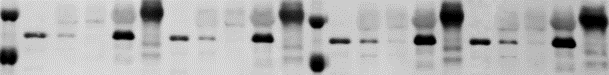


**50Kda**

**WB:TDP43**

**WB:Ataxin2**

**150Kda**

**Figure 1B original:**

**WB:Ataxin2**

**Upper Panel:**


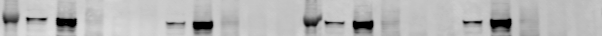


**Duplication of 2 indepenent animals Duplication of 2 indepenent animals**


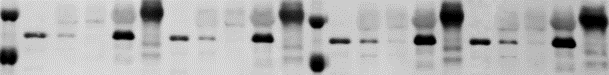
 **Lower Panel:**

**WB:TDP43**

**Duplication of 2 indepenent animals Duplication of 2 indepenent animals**

**Figure 3B:**


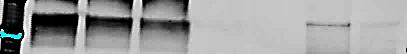

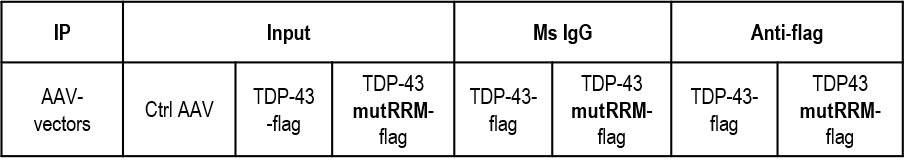

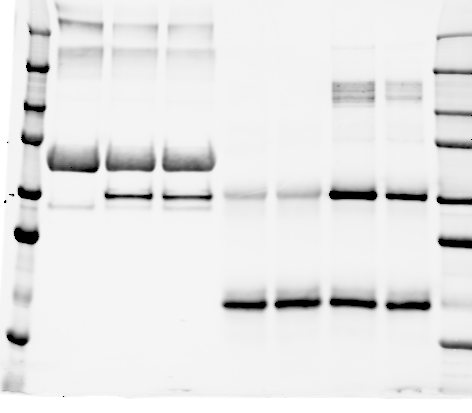


**150KD**

**50KD**

**WB:TDP43**

**WB:Ataxin-2**

**Figure 3B original:**


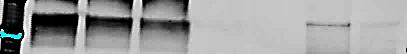

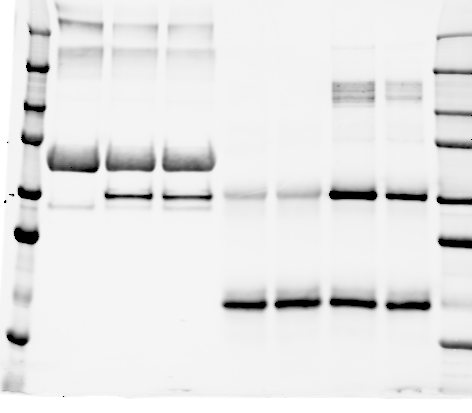


**WB:TDP43**

**WB:Ataxin-2**

**Figure 5B:**

**150Kda**

**50Kda**

**37Kda**

**50Kda**

**37Kda**

**ATXN2**

**TDP43-flag**

**TDP43-flag**


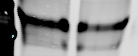


**AAV8-TDP43-flag: ― +**


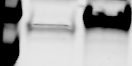


**TDP43 (endogenous)**


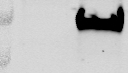

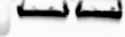


**37Kda**

**β-actin**

**Figure 5B non-cropped:**


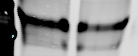


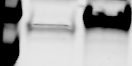


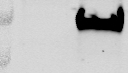


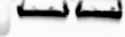


**Detailed data analysis description for supplementary IP-MS data:**

Human iPSC-derived GABA neurons were infected with either AAV-ctrl or AAV-TDP-43, and the lysate was immunoprecipitated with either isotype IgG control or Ataxin-2 antibody with 3 biological replicates. The elutes were followed by tryptic digestion and LC-SWATH MS analysis with 3 technical replicates to identify binding cofactors of TDP-43 overexpression vs control (endogenous TDP-43).

For LC-SWATH MS analysis, the DIA samples, spectral alignment and targeted data extraction were carried out using the SWATH Processing Micro App in Peakview software (Version 1.2, Sciex). These analyses were performed using the reference spectral library that was previously generated. For each comparison group, four DIA raw files were simultaneously loaded with an extraction window of 20 minutes. The parameters used for data analysis included the selection of 5 peptides, 8 transitions, and a minimum peptide confidence level of 99%. These parameters considered shared peptides and set the XIC (extracted ion chromatogram) width at 50 ppm. Following data processing, the processed mrkvw files that contained protein information obtained from PeakView were imported into MarkerView software (Version 1.2.1, Sciex). The protein intensities (peak areas) for all runs were normalized using the built-in total ion intensity sum plug-in. Prior to subsequent statistical analysis, a Log2 transformation was applied to the data. The adjusted p-values for each protein were calculated using an unpaired t-test, without assuming a consistent standard deviation. To control false discoveries, a two-stage step-up method of Benjamini, Krieger, and Yekutieli with a Q-value of 5% was employed. Differentially expressed proteins are defined by Log2 (Fold change) >1 and -Log10 (adjusted P) >1.301.

As for the Ingenuity pathway analysis, all TDP-43 interacting proteins of the TDP-43 vs Normal comparison group were separately analyzed in the IPA software version 24.0.1 (QIAGEN Inc.). The gene symbols and corresponding expression fold change values of the identified proteins were inputted into IPA (Ingenuity Pathway Analysis). The Core Analysis function was executed with the following parameters: fold change in expression was selected as the analysis type, networks were generated considering both direct and indirect relationships, and the confidence level was restricted to experimentally observed data for the relationships. The cut-off values applied to all datasets included fold change ≥1.5 for up-regulated and ≤-1.5 for down-regulated proteins. Adjusted p values (Benjamini-Hochberg, FDR) of <0.05 were considered significant. Based on the IPA's analysis, significant canonical pathways, biological functions and diseases, and interaction networks were algorithmically generated.
